# Crystal and Dislocation Characteristics of Ti-6Al-4V Alloy Under Effect of Laser Shock Peening

**DOI:** 10.3390/ma18020378

**Published:** 2025-01-15

**Authors:** Cheng Gu, Chun Wang, Jianhua Zhao, Yajun Wang, Zenghui Tian

**Affiliations:** 1 College of Materials Science and Engineering, Chongqing University, Chongqing 400045, China; gucheng.90@cqu.edu.cn (C.G.);; 2 National Engineering Research Center for Magnesium Alloys, Chongqing University, Chongqing 400044, China; 3 National Key Laboratory of Advanced Casting Technologies, Chongqing University, Chongqing 400044, China; 4 Henan Zhongyuan Special Steel Equipment Manufacturing Co., Ltd., Jiyuan 459099, China

**Keywords:** laser shock peening, microstructure, crystallographic characteristics, dislocation, Ti-6Al-4V alloy

## Abstract

Laser shock peening (LSP) is an effective method for enhancing the fatigue life and mechanical properties of Ti alloys. However, there is limited research on the effects of LSP on crystal structure and dislocation characteristics. In this study, Ti-6Al-4V alloy was subjected to laser shock peening with varying laser power levels. The influence of laser power on the microstructure of Ti-6Al-4V was investigated, with a focus on the evolution of the cross-sectional structure, crystallographic features, and dislocation behavior. These characteristics were analyzed using scanning electron microscopy (SEM), electron backscatter diffraction (EBSD), and transmission electron microscopy (TEM). Following laser shock peening, the surface grains of Ti-6Al-4V alloy exhibited a distinct preferred orientation and underwent significant refinement, resulting in the formation of nanocrystals. At a laser power of 8 J, the texture strength decreased to 5.19 mud. As laser power increased, a denser dislocation structure and high-density dislocation regions formed at the surface, and the subgrain size further decreased, reaching 66 nm at 8 J. These findings provide valuable insights into grain refinement and property enhancement, contributing to the understanding of process–microstructure–property relationships.

## 1. Introduction

Ti-6Al-4V alloy, a typical α+β dual-phase titanium alloy, is widely used in the aerospace and medical fields and other industries due to its high specific strength, excellent corrosion resistance, and stable physical and chemical properties [1,2,3]. However, its relatively low tribological properties and fatigue resistance present challenges in meeting the long-term performance demands of critical components in harsh service environments [4,5,6]. To address these issues, various surface modification techniques have been employed to enhance the mechanical properties of Ti alloys without altering the matrix material. These include shot peening (SP) [7,8], surface mechanical attrition treatment (SMAT) [9], deep cold rolling (DCR) [10], and laser shock peening (LSP) [11]. Laser shock peening (LSP) is one of the most effective surface treatment methods for improving the fatigue life and mechanical properties of metal alloys [12,13]. During LSP, the metal surface experiences severe deformation induced by ultra-high strain rates (>10^6^ s^−1^) generated by high-pressure shock waves (~GPa) and ultra-short laser pulses (10–30 ns) [14,15,16]. This process produces significant strengthening effects, making LSP a popular technique for enhancing the service life of structural materials without altering the base material [17,18].

Numerous studies have been conducted to improve the fatigue resistance [19,20,21], corrosion resistance [22,23,24,25], and wear resistance [26,27] of Ti alloys through LSP. Jia et al. [28] investigated the changes in microhardness and residual stress distribution in a near-α Ti alloy following LSP treatment. Lin et al. [29] demonstrated that increasing the laser shock time or energy significantly enhanced the microhardness of Ti alloys. Madapana et al. [30] observed that LSP treatment refined the surface grains of Ti alloys, while the surface roughness increased with higher laser power. In addition to the improvements in the properties of Ti alloys, the effects of LSP on microstructure have also been studied. Lainé et al. [7] found that LSP induced directional planar dislocations and the formation of dislocation cell networks and subgrains. Guo et al. [31] observed a high density of dislocation lines, tangles, and multi-directional mechanical twins after LSP and suggested that grain refinement was responsible for the enhancements in yield strength, ultimate tensile strength, and elongation following the treatment. Li et al. [19] proposed the coupling strengthening process of laser shock peening assisted by cryogenic temperature for the problem of high-cycle bending fatigue (HCBF) of aeroengine blades, and found that higher-density dislocation, deformation twins, and high-amplitude compressive residual stress can suppress the initiation and propagation of HCBF cracks, thus significantly improving the HCBF life. Bai et al. [32] found that microstructures formed by laser shock peening lead to higher microhardness, corrosion resistance, and fatigue life, which are significantly beneficial for preventing life the cycle failure of mega-scale engineering structures in critical environments. Most studies are focused on the properties of the laser shock-treated materials [33,34,35].

Numerous studies have been conducted to enhance the fatigue resistance [19,20,21], corrosion resistance [22,23,24,25], and wear resistance [26,27] of Ti alloys through LSP. Jia et al. [28] investigated the changes in microhardness and residual stress distribution in a near-α Ti alloy following LSP treatment. Lin et al. [29] demonstrated that increasing the laser shock time or energy improved the microhardness of Ti alloys. Madapana et al. [30] observed that LSP treatment refined the surface grains of Ti alloys, although surface roughness increased with higher laser power. In addition to improving the properties of Ti alloys, the effects of LSP on microstructure have also been studied. Lainé et al. [7] found that LSP induced directional planar dislocations and the formation of dislocation cell networks and subgrains. Guo et al. [31] observed a high density of dislocation lines, tangles, and multi-directional mechanical twins after LSP, suggesting that grain refinement was responsible for the observed improvements in yield strength, ultimate tensile strength, and elongation. Li et al. [19] proposed a coupled strengthening process of LSP assisted by cryogenic temperature to address high-cycle bending fatigue (HCBF) in aeroengine blades. They found that the higher density of dislocations, deformation twins, and high-amplitude compressive residual stresses could suppress the initiation and propagation of HCBF cracks, significantly improving HCBF life. Bai et al. [32] demonstrated that microstructures formed by LSP enhanced microhardness, corrosion resistance, and fatigue life, which are beneficial for preventing life cycle failure in mega-scale engineering structures operating in critical environments. Most research to date has focused on the properties of LSP-treated materials [33,34,35].

To elucidate the mechanism behind property improvements induced by LSP, studies have also focused on local deformation [14] and residual stress [36,37,38]. Peyre et al. [39] reviewed the basic physical processes involved in LSP and analyzed the pressure loadings generated under different laser conditions. Zhao et al. [40] investigated the effects of various laser shock paths on deformation and residual stress distribution in materials. Sun et al. [41] found that LSP introduced compressive residual stresses greater than 200 MPa. In a previous study by the authors [42], the effect of LSP on stress wave evolution was examined through multi-scale simulations, revealing that both elastic and plastic deformations occur under the high pressures induced by LSP. While extensive research has been conducted on the effects of LSP on mechanical properties [43,44,45], there has been limited investigation into the characteristics and evolution of crystals and dislocations at the micro-scale in Ti alloys during LSP. Given that Ti-6Al-4V alloy contains both α and β phases, which may interact during LSP, understanding these interactions is crucial. Such studies are essential for establishing process–microstructure–property relationships.

In this study, Ti-6Al-4V alloy was subjected to LSP at varying laser power levels. The effects of LSP on the cross-sectional microstructure, crystallographic characteristics, and dislocation behavior of the alloy were investigated and discussed. A mechanism for the microstructural evolution of Ti-6Al-4V during LSP was proposed, aiming to enhance the understanding of grain refinement and the property improvements resulting from LSP treatment.

## 2. Materials and Methods

Ti-6Al-4V alloy with the chemical composition shown in Table 1 was used in this study. The Ti-6Al-4V samples, in their as-cast state, were first rolled at 920 °C, heat-treated at 750 °C for 1 h, and then air-cooled. The surfaces of the samples to be treated by LSP were ground and ultrasonically cleaned using a solution of acetone and ethanol. For the LSP experiment, a YS100-R200A Nd:YAG laser was employed. A piece of 100 μm thick black tape was used as the energy absorption layer, and a 2 mm thick water flow layer served as the confinement layer. The experiment was conducted with laser powers of 6 J, 7 J, and 8 J, respectively. Varying laser powers resulted in different degrees of plastic deformation on the Ti-6Al-4V surface, significantly influencing its material properties. Other experimental parameters included a pulse duration of 20 ns, a pulse wavelength of 1064 nm, a pulse frequency of 10 Hz, an overlap ratio of 50%, and a spot diameter of 3 mm. The laser power density can be calculated as follows [46]:

(1) $$I_{0}=\frac{4E}{\pi D^{2}\tau }$$

where *E* is the laser power, *D* is the spot diameter, and *τ* is the pulse duration. Accordingly, laser powers of 6 J, 7 J, and 8 J produce laser power densities of 4.25, 4.95, and 5.65 GW/cm^2^, respectively, which exceed the dynamic strain aging threshold of Ti-6Al-4V alloy. Additional details can be found in the authors’ previous publications [27,42].

The LSP-treated samples were sectioned to dimensions of 20 × 10 × 2 mm^3^ using electric discharge machining (EDM). The cross-sections were then polished, etched with Kroll’s reagent, and cleaned with absolute ethanol. The cross-sectional microstructure at the surface was examined and analyzed using a scanning electron microscope (SEM, TESCAN VEGA III). Electron backscatter diffraction (EBSD, EDAX-TSL) was employed to obtain crystallographic information, such as grain size, grain orientation, etc. The EBSD scanning area covered a 300 μm × 300 μm region of the cross-section along the depth direction of the laser-shocked surface. Transmission electron microscopy (TEM, Talos F200X, Thermo Fisher Scientific, Waltham, MA, USA) was used to observe nanoscale features such as dislocations, twins, and grain boundaries. The sample for TEM analysis was prepared using focused ion beam (FIB, FEI Scios 2 HiVac, Thermo Fisher Scientific, MA, USA) technology.

## 3. Results and Discussion

### 3.1. Cross-Sectional Microstructure

**Figure 1:**
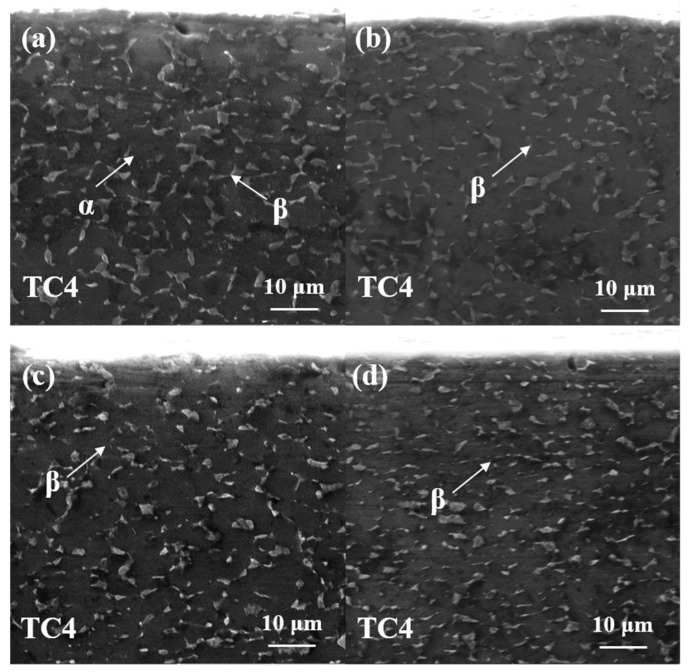
Microstructure of TD section of Ti-6Al-4V alloy treated with different laser powers of (**a**) 0 J, (**b**) 6 J, (**c**) 7 J, and (**d**) 8 J.

During the rolling process, the material exhibits varying properties depending on the direction. In the transverse direction (TD) to the rolling direction, the grains are typically stretched and flattened, leading to increased strength and hardness. In the longitudinal direction (LD) to the rolling direction, the grains tend to be more elongated and aligned, resulting in improved ductility and plasticity. The cross-sectional microstructures of Ti-6Al-4V alloy treated with different laser powers in the TD are shown in Figure 1.

As depicted in Figure 1a, the cross-section of the Ti-6Al-4V alloy displays a typical α+β dual-phase microstructure. The light gray α phase, which forms the substrate, exhibits a coarse, equiaxed crystal structure, while the bright white β phase is dispersed, with a smaller volume fraction than the α phase, and shows an equiaxed-to-elongated morphology. As shown in Figure 1b–d, after laser shock peening treatment, the β phase undergoes refinement, with some β phase regions transitioning from elongated to short rod-like or equiaxed shapes (as indicated by arrows). Nanoscale β phase grains can also be observed. Additionally, the distribution of the β phase within the substrate becomes more uniform after laser shock peening, which can help prevent crack propagation. Compared to the untreated samples, the number of small β phase regions increases. As the laser power increases, the β phase morphology becomes more equiaxed, and the number of nanocrystals also increases correspondingly.

**Figure 2:**
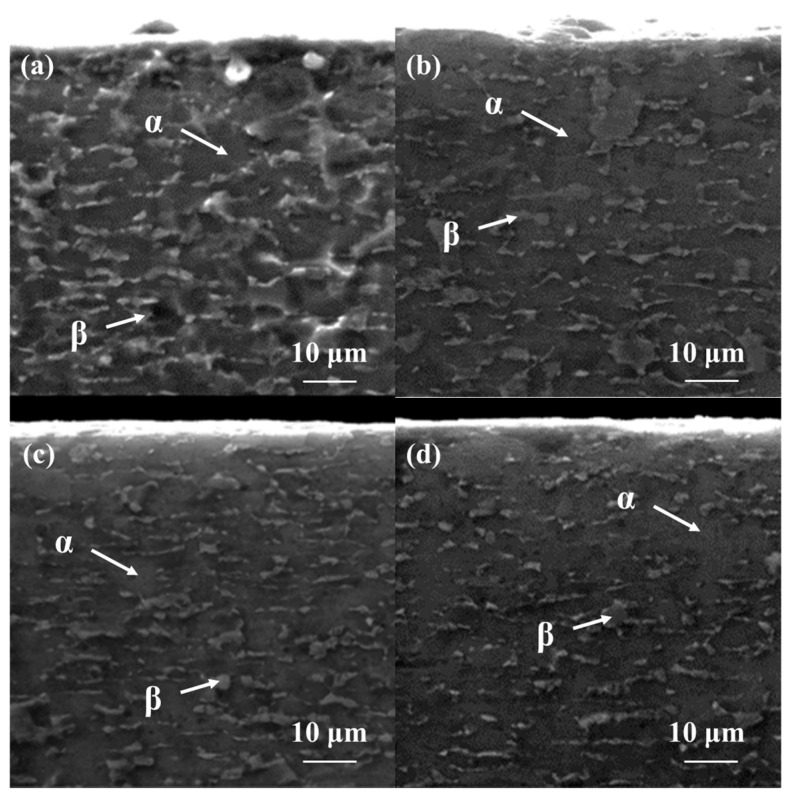
Microstructure of LD section of Ti-6Al-4V alloy treated with different laser powers of (**a**) 0 J, (**b**) 6 J, (**c**) 7 J, and (**d**) 8 J.

The cross-sectional microstructure of Ti-6Al-4V alloy in the LD, treated with different laser powers, is shown in Figure 2. Compared to the TD, the β phase in the LD exhibits a more elongated shape and is laterally distributed. This is attributed to the grain elongation and directional deformation caused by the compressive forces during rolling. The Ti-6Al-4V sample without laser shock peening treatment (0 J) shows a larger volume of β phase, with some elongated β phases displaying interconnected characteristics. As shown in Figure 2b–d, after laser shock peening, the β phase in the LD also undergoes refinement, transitioning from larger β phase regions to equiaxed and needle-like structures (as indicated by the arrows). With increasing laser power, the proportion of β phase grains rises, and the refinement effect becomes more pronounced.

As mentioned above, significant changes in the microstructure were observed in both the TD and LD cross-sections after laser shock peening treatment. During the laser shock peening process, the surface of the Ti-6Al-4V titanium alloy is subjected to intense laser-induced shock waves, which induce strong plastic deformation of the material’s surface. This results in alterations to both the morphology and microstructure of the material. Additionally, residual compressive stresses of varying amplitudes are generated within the material due to rapid cooling and uneven heat transfer during the process [32,47]. The formation of residual compressive stresses can influence the growth mechanism of the β phase, effectively inhibiting its expansion. In addition, laser shock peening induces lattice distortion and promotes dislocation proliferation on the material’s surface, which, in turn, enhances the refinement and nucleation of the β phase. As a result, after laser shock peening, the β phase typically exists in a finer form. The presence of smaller β phase grains improves the overall performance of titanium alloys, enhancing properties such as hardness, wear resistance, oxidation resistance, and corrosion resistance, ultimately extending the material’s service life.

### 3.2. Crystallographic Characteristics

To investigate the effect of laser shock peening on the surface grain size and orientation of Ti-6Al-4V alloy, EBSD was employed to characterize and analyze the crystallographic characteristics of both treated and untreated samples. After laser shock peening, it was observed that the change in grain orientation in the TD was relatively minor. As a result, the microstructure in the LD of the Ti-6Al-4V alloy was further examined to analyze grain orientation and grain boundary characteristics.

**Figure 3:**
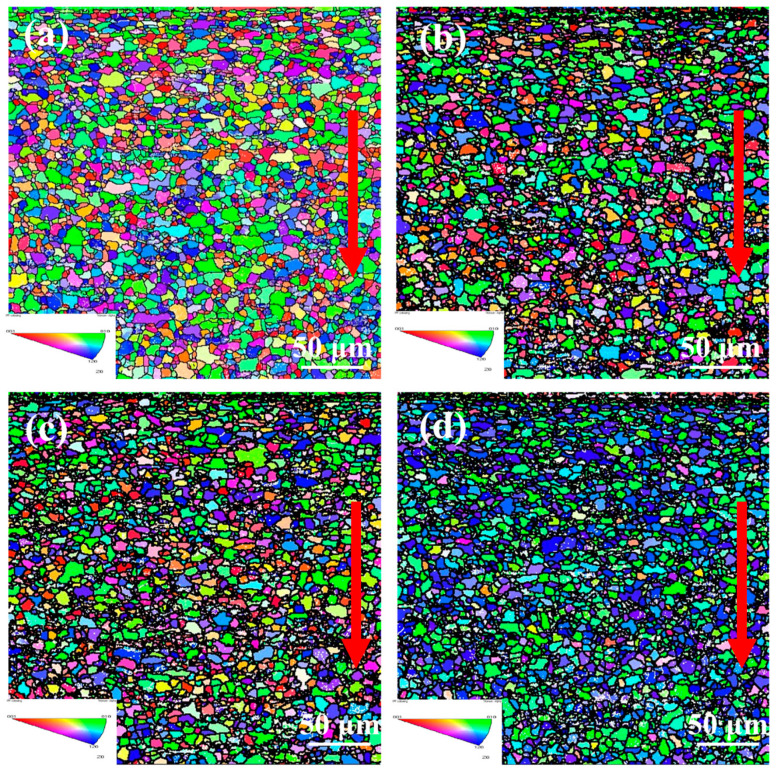
Inverse pole figure of Ti-6Al-4V alloy treated with different laser powers of (**a**) 0 J, (**b**) 6 J, (**c**) 7 J, and (**d**) 8 J. (The red arrow means the depth direction from the Ti-6Al-4V surface).

Figure 3 shows a reverse pole diagram of Ti-6Al-4V alloy treated with different laser powers. The various colors in the diagram represent different grain orientations, with the direction indicated by the arrow corresponding to the depth direction from the Ti-6Al-4V surface. It can be observed that after laser shock peening, there is a noticeable change in the color of the surface grains, indicating that the grains begin to align along a specific direction, resulting in a preferred orientation. This alignment occurs due to the uneven plastic deformation on the surface of the Ti-6Al-4V during laser shock peening. Crystals with a higher density of slip systems are more prone to plastic deformation, leading to more pronounced grain orientations, while certain crystal planes, with lower energy, are more likely to form, influencing the final orientation outcome.

From Figure 3a, it can be seen that the surface of the untreated sample features elongated grains, with equiaxed grains beneath. After laser shock peening, the surface grains are refined, resulting in the formation of nanocrystals, and the grain size distribution along the depth direction becomes more uniform, as shown in Figure 3b–d. As the laser power increases, the depth of surface grain refinement also increases. The areas with significant surface grain refinement reach depths of 12.93 μm, 16.38 μm, and 22.41 μm, respectively. Overall, the surface grains of the material undergo refinement, and some of the grains within the material also experience refinement. The equiaxed grain size in the treated sample is smaller than that of the untreated sample.

**Figure 4:**
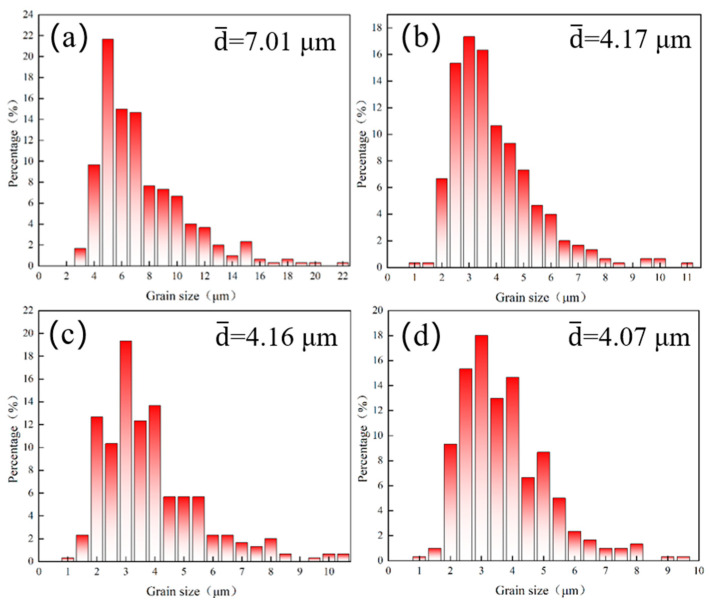
Grain size distribution of Ti-6Al-4V alloy in the surface layer of 50 μm under different laser powers of (**a**) 0 J, (**b**) 6 J, (**c**) 7 J, and (**d**) 8 J.

Figure 4 shows the grain size distribution within a depth range of 50 μm from the surface. The average grain size of the untreated sample (0 J) is 7.01 μm, with most of the grains ranging from 4 to 10 μm as shown in Figure 4a. This is due to the generally small surface grain size of the sample after rolling treatment. After laser shock peening with laser powers of 6 J, 7 J, and 8 J, as shown in Figure 4b–d, the average grain size of the surface layer decreased to 4.17 μm, 4.16 μm, and 4.07 μm, respectively. As seen in Figure 3, the surface grain size of the sample decreases significantly after laser shock peening. However, when considering the numerical values in Figure 4, the reduction in grain size appears modest, with the average grain sizes at the surface under different laser power levels being nearly identical. This can be attributed to the formation of a thin layer of nanocrystals on the surface after laser shock peening, which were not fully captured in the statistical analysis due to their small size. The grain size distribution reveals that the surface grain size is generally refined to the range of 10 μm following laser shock peening. As the laser power increases, the proportion of surface grains in the 2–5 μm range gradually increases, indicating that the degree of grain refinement also improves.

**Figure 5:**
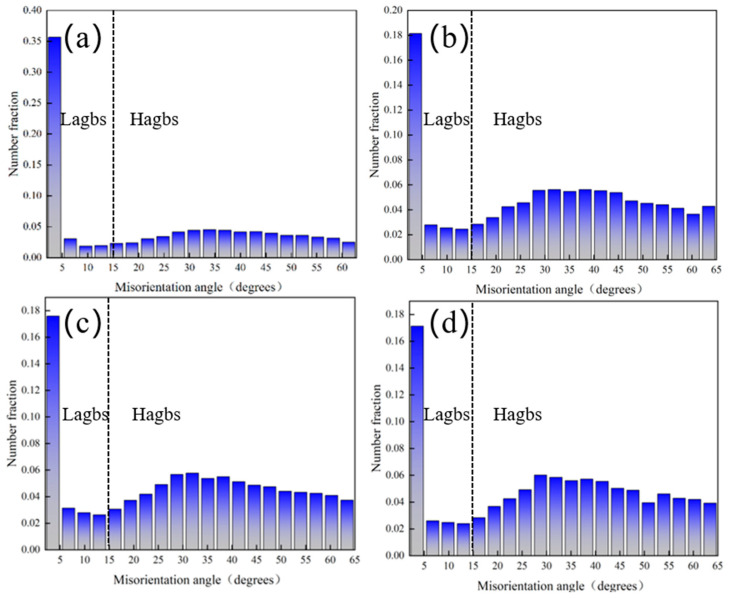
Histogram of orientation difference frequency distribution of Ti-6Al-4V alloy treated with different laser powers of (**a**) 0 J, (**b**) 6 J, (**c**) 7 J, and (**d**) 8 J.

Figure 5 shows a histogram of the orientation difference frequency distribution for Ti-6Al-4V alloy treated with different laser powers. Grain boundaries are typically classified into large-angle grain boundaries (HAGBs > 15°) and small-angle grain boundaries (2° < LAGBs < 15°), based on the magnitude of the phase difference angle. As shown in Figure 5a, the proportion of small-angle grain boundaries in the original Ti-6Al-4V alloy is 36.3%. After laser shock peening treatment, the proportion of small-angle grain boundaries decreases, while the proportion of large-angle grain boundaries increases. As the laser power increases, the proportion of small-angle grain boundaries gradually declines. Specifically, as shown in Figure 5b–d, the proportion decreases to 21.2%, 21%, and 20.4%, respectively, while the proportion of large-angle grain boundaries increases by 15.1%, 15.3%, and 15.9%, respectively. During the laser shock peening process, severe plastic deformation on the surface induces grain rotation and rearrangement, leading to grain refinement and an increase in grain boundary angle. Additionally, the high temperature and pressure conditions during laser shock peening promote dynamic recrystallization, allowing new grains to nucleate and grow along existing grain boundaries, which further increases the grain boundary angle. Moreover, large-angle grain boundaries have higher energy than small-angle boundaries, making them more readily formed during laser shock peening, which ultimately reduces the number of small-angle grain boundaries and increases the number of large-angle grain boundaries on the surface. This result is consistent with Yang’s study [48], which also observed a decrease in the proportion of small-angle grain boundaries following laser shock peening.

**Figure 6:**
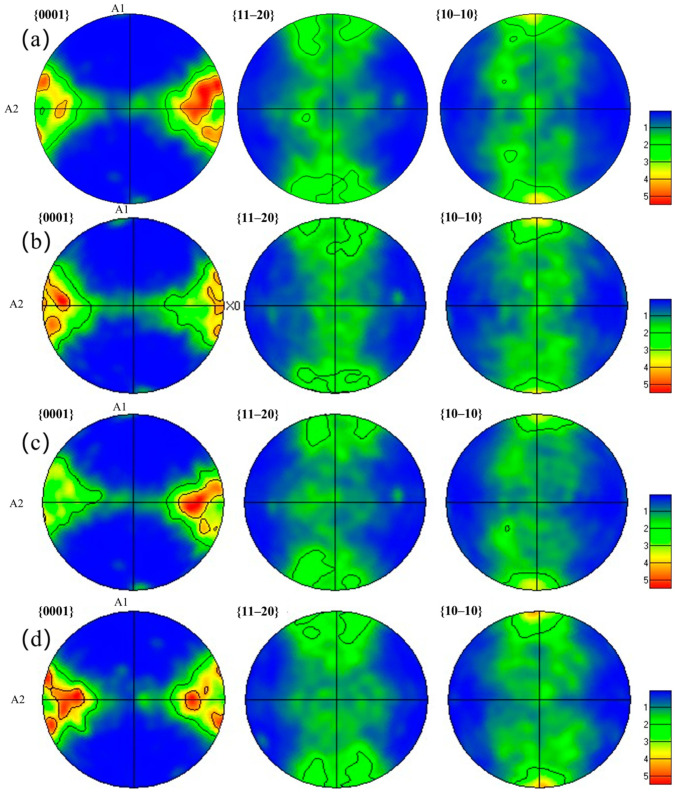
α-phase polar diagram of Ti-6Al-4V alloy treated with different laser powers of (**a**) 0 J, (**b**) 6 J, (**c**) 7 J, and (**d**) 8 J.

Figure 6 presents an α-phase pole diagram of Ti-6Al-4V alloy subjected to different laser powers, where A1 represents the vertical rolling direction and A2 represents the parallel rolling direction. As shown in Figure 6a, the substrate exhibits a weak texture, with a maximum texture strength of 5.51 mud. After laser shock peening treatments with laser powers of 6 J, 7 J, and 8 J, the texture strength slightly decreases to 5.43 mud, 5.26 mud, and 5.19 mud, respectively, as shown in Figure 6b–d. Laser shock peening induces grain refinement and the formation of new grain boundaries in the surface layer of the sample, disrupting the original orientation texture. Additionally, grain rearrangement and an increase in dislocation density lead to a more uniform grain orientation distribution. The occurrence of dynamic recrystallization further weakens the texture characteristics of the surface layer, which results in a reduction in the polar density of specific crystal planes in the pole diagram.

### 3.3. Dislocation Behavior

**Figure 7:**
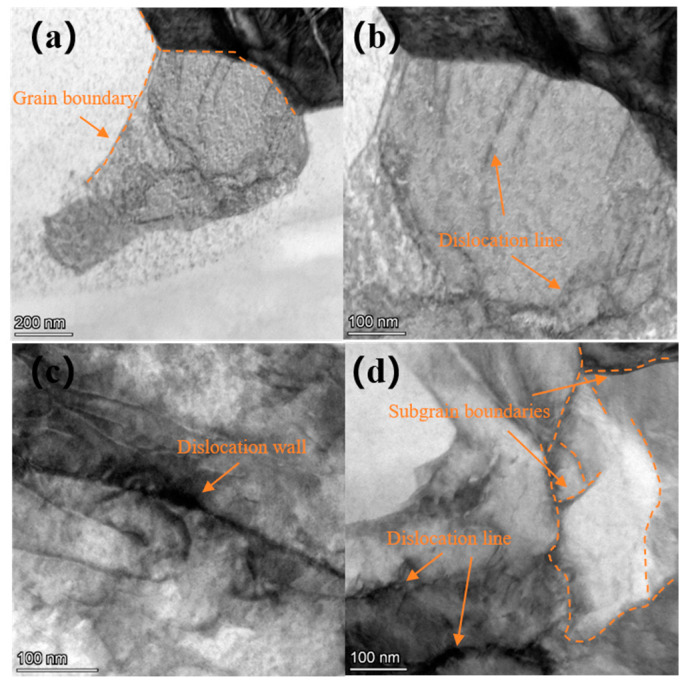
TEM image of Ti-6Al-4V substrate surface layer: (**a**,**b**) the microstructure of the β phase, and (**c**,**d**) the microstructure of the α phase.

To examine the dislocation characteristics of the treated surface, TEM was employed. A TEM image of the surface layer of Ti-6Al-4V alloy is shown in Figure 7. Figure 7a,b display the microstructure of the β phase, where only a few scattered dislocation structures are observed, and the boundaries of the β phase are clearly visible prior to laser shock peening. Figure 7c,d show the microstructure of the α phase, where dislocation lines and dislocation walls are evident, along with a higher dislocation density compared to the β phase. Additionally, a small amount of subgrain boundary formation can be observed.

**Figure 8:**
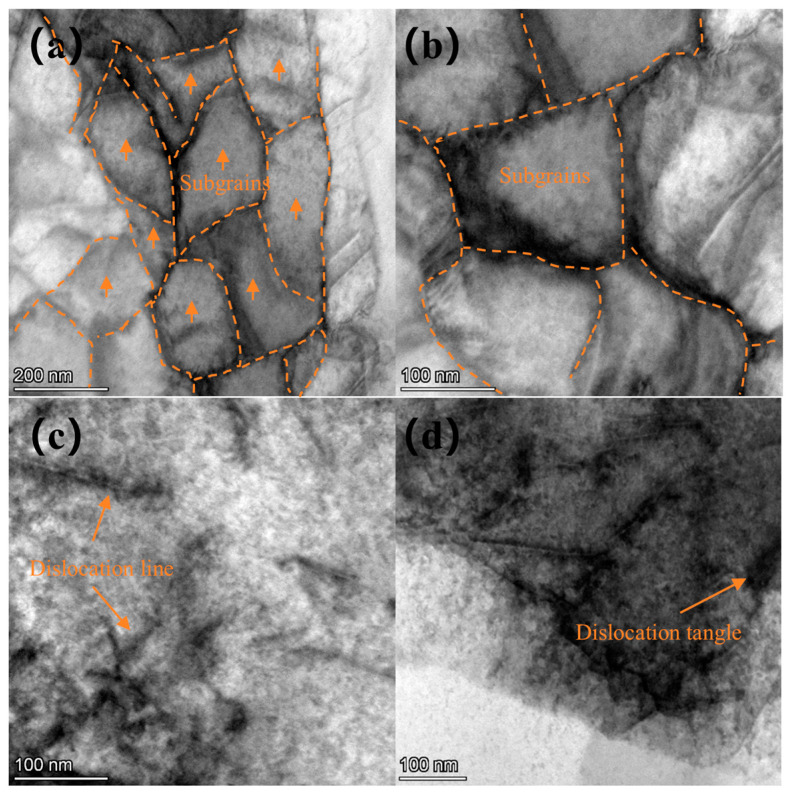
TEM image of Ti-6Al-4V sample surface under laser power of 6 J: (**a**) subgrains, (**b**) subgrains at higher magnification, (**c**) dislocation line, and (**d**) dislocation tangle.

A TEM image of the surface layer of Ti-6Al-4V alloy subjected to a laser power of 6 J is shown in Figure 8. It is evident that dislocations accumulate continuously, gradually forming structures such as dislocation walls and entanglements on the surface. The plastic deformation induced by laser shock peening leads to the accumulation of surrounding dislocations, which migrate towards the dislocation walls under applied stress. The image reveals dislocation wall structures in multiple directions, and the grains are subdivided into smaller subgrain structures by intersecting dislocation walls, with a subgrain size of 211 nm. A small amount of dislocation entanglement is also observed within the crystal structure. Compared to the untreated substrate, the dislocation density on the surface of the sample after laser shock peening is significantly increased.

**Figure 9:**
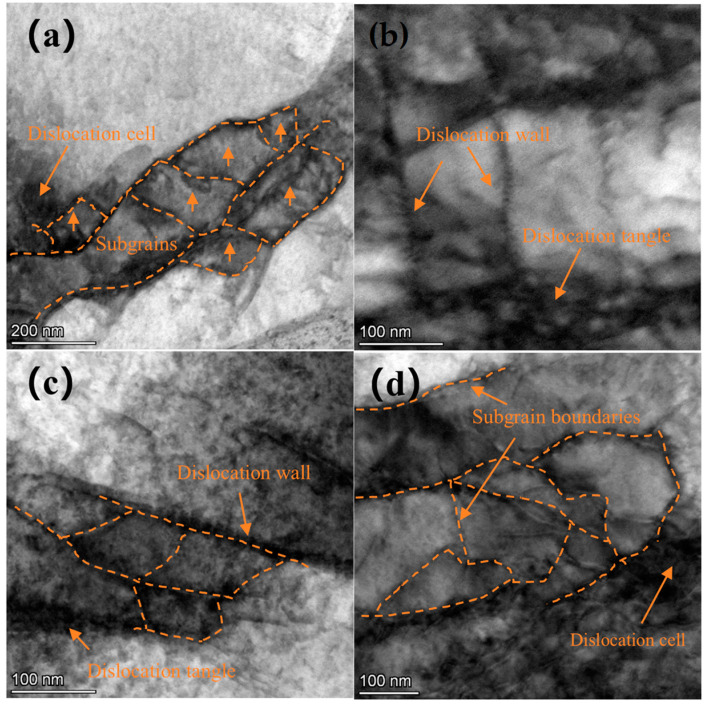
TEM image of Ti-6Al-4V sample surface under laser power of 7 J: (**a**) subgrains, (**b**,**c**) dislocation wall, and (**d**) subgrains at higher magnification.

Figure 9 shows a TEM image of the surface layer of the Ti-6Al-4V sample subjected to a laser power of 7 J. Similarly to the 6 J sample, structures such as dislocation walls, dislocation entanglements, and subgrains are observed, along with a small number of dislocation cells. Compared to the sample treated with laser power of 6 J, more pronounced dislocation characteristics are evident, and the dislocation density increases. The dislocations surrounding the dislocation walls undergo annihilation and rearrangement, leading to the formation of subgrain boundaries. The accumulation of dislocations near these boundaries results in an increased orientation difference around the grain boundaries. Ultimately, the grains are subdivided into several equiaxed, refined subgrains, with a subgrain size of 134 nm.

**Figure 10:**
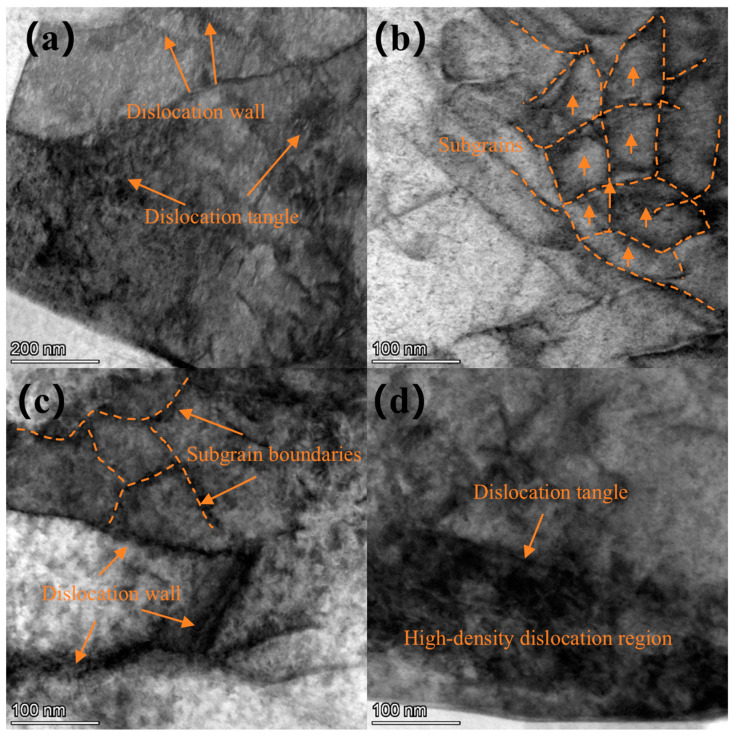
TEM image of Ti-6Al-4V sample surface under laser power of 8 J: (**a**) dislocation tangle, (**b**,**c**) subgrains, and (**d**) dislocation tangle at higher magnification.

Figure 10 shows a TEM image of the surface layer of the Ti-6Al-4V alloy treated with a laser power of 8 J. Dense dislocation lines can be observed on the surface, forming more dislocation tangles, dislocation walls, and subgrains. The subgrain size of the 8 J sample is 66 nm. High-density dislocation regions are present on the surface, with the dislocation density further increased compared to the 7 J sample. It can be concluded that laser shock peening significantly alters the grain structure, dislocation characteristics, and other properties at the surface of Ti-6Al-4V. The high-pressure shock wave generated during the laser shock peening process induces severe plastic deformation on the material surface, promoting the proliferation and movement of dislocations. Under the 6 J laser treatment, the material’s surface crystal structure absorbs energy, leading to the proliferation and continuous aggregation of dislocations, which form dislocation walls and entanglement structures, while also facilitating the nucleation and growth of nanoscale subgrains. As the laser power increases, the degree of plastic deformation becomes more intense, resulting in denser dislocation entanglements, dislocation walls, and high-density dislocation regions on the surface, further reducing the subgrain size.

### 3.4. Microstructure Evolution Mechanism

**Figure 11:**
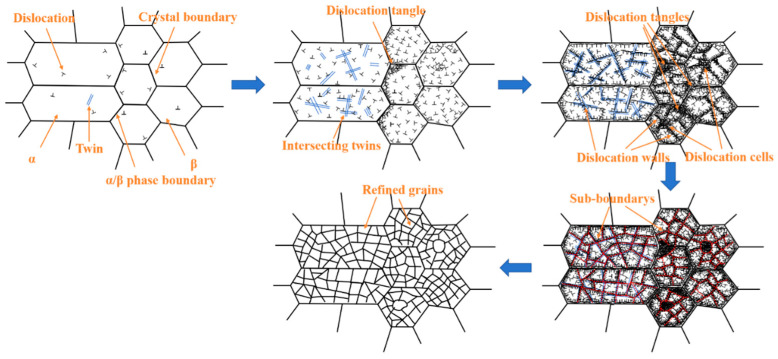
Schematic diagram of microscopic evolution mechanism at the surface layer of Ti-6Al-4V alloy under laser shock peening.

Based on the microstructure, crystallography, and dislocation evolution described above, it is evident that a significant number of dislocation defects were generated on the surface of Ti-6Al-4V after laser shock peening treatment. These dislocations were subjected to multi-directional impact loads during the slip process, gradually evolving into a variety of complex microstructures, including dislocation walls, dislocation cells, and dislocation entanglements. The formation of these structures played a key role in promoting grain refinement. Ao et al. [49] and Ren et al. [50] demonstrated that when laser shock peening induces shock waves into the interior of Ti-6Al-4V alloy, these shock waves interact with the surface lattice structure, causing atomic misalignment and the formation of high-density dislocations and twins within the crystal. The interaction and dislocation movement between the α phase and multi-directional twin crystals lead to grain refinement, while the β phase undergoes refinement through the sliding, stacking, entanglement, and rearrangement of numerous dislocations. Based on these observations, a schematic model illustrating the microstructure evolution of the surface layer of Ti-6Al-4V alloy under laser shock peening is presented in Figure 11.

The microstructure evolution of the Ti-6Al-4V surface induced by laser shock peening treatment can be divided into the following stages:

1. Before laser shock peening, a small number of dislocations and twins are randomly distributed within the α and β phases.
2. During laser shock peening, severe plastic deformation occurs on the surface of the Ti-6Al-4V alloy. Due to stress concentration at the grain boundaries, dislocations are more likely to form at both grain and phase boundaries. Twinning becomes more pronounced in the α phase, and multi-directional loading causes the intersection and interaction of twins. Meanwhile, dislocations continue to proliferate and slip towards the twins. Inside the β phase and at the grain boundaries, dislocations are generated, proliferating and migrating.
3. As deformation intensifies, twinning in the α phase forms and expands in multiple directions. High-density dislocations begin to accumulate near the twins. In the β phase, dislocation slip is hindered by grain boundaries, causing dislocations to accumulate and entangle near these boundaries, gradually forming dislocation walls, dislocation tangles, and dislocation cells.
4. With continued plastic deformation, the dislocation density increases further. Twin crossing and dislocation interactions within the α phase lead to the formation of blocky subgrains. In the β phase, dislocations are compressed and accumulated towards dislocation walls and dislocation cells. To minimize the total energy of the system, dislocations around these structures undergo annihilation and rearrangement, forming subgrain boundaries.
5. Dislocations continue to accumulate near the subgrain boundaries, increasing the orientation differences around the grain boundaries. This increase in orientation difference leads to the segmentation and refinement of the original grains.

## 4. Conclusions

In this study, we investigated the effect of laser power on the microstructure of Ti-6Al-4V alloy through laser shock peening (LSP) treatment, focusing on the evolution of the cross-sectional structure, crystallographic characteristics, and dislocation behavior. After LSP treatment, the surface grains of Ti-6Al-4V alloy exhibited a distinct preferred orientation and underwent significant refinement, leading to the formation of nanocrystals. The equiaxed grain size in the treated sample was smaller than that in the untreated sample. As laser power increased, both the degree of grain refinement and the thickness of the nanocrystalline layer increased. At a laser power of 8 J, the average grain size of the surface layer was 4.07 μm, and the thickness of the nanocrystalline layer reached 22.41 μm.

After LSP treatment, the proportion of small-angle grain boundaries decreases, and the texture strength slightly weakens. As laser power increases, both the proportion of small-angle grain boundaries and the texture strength decrease accordingly. Specifically, as laser power increases from 6 J to 7 J and 8 J, the proportion of small-angle grain boundaries decreases from 21.2% to 21% and 20.4%, respectively, while the proportion of large-angle grain boundaries increases from 15.1% to 15.3% and 15.9%, respectively. At a laser power of 8 J, the texture strength decreases from 5.51 mud to 5.19 mud. LSP promotes the proliferation and movement of dislocations on the surface of Ti-6Al-4V alloy, as well as the nucleation and growth of subgrains. This leads to the formation of structures such as dislocation walls, dislocation cells, dislocation entanglements, and subgrains. As laser power increases, a denser dislocation structure and high-density dislocation regions form on the surface, and the subgrain size decreases further, reaching 66 nm at a laser power of 8 J.

## Figures and Tables

**Table 1:** Chemical composition of Ti-6Al-4V alloy used in this study (wt%).

Element	Al	V	Fe	C	N	H	O	Ti
Content	6.26	3.81	0.23	0.04	0.03	0.01	0.12	Bal.

## Data Availability

All data that support the findings of this study are available from the corresponding author upon reasonable request.
